# Re-using questions in classroom-based assessment: An exploratory study at the undergraduate medical education level

**DOI:** 10.1007/s40037-018-0482-1

**Published:** 2018-11-12

**Authors:** Sébastien Xavier Joncas, Christina St-Onge, Sylvie Bourque, Paul Farand

**Affiliations:** 1 Hôtel-Dieu de Québec, Québec, Canada; 2 Department of Medicine, Faculty of Medicine and Health Sciences, Université de Sherbrooke, Québec, Canada; 3 Undergraduate Medical Education Program, Faculty of Medicine and Health Sciences, Université de Sherbrooke, Québec, Canada; 4 Department of Medicine, Faculty of Medicine and Health Sciences, Université de Sherbrooke, Québec, Canada

**Keywords:** Classroom-based assessment, Item banks, Psychometric properties, Re-using questions

## Abstract

**Introduction:**

To alleviate some of the burden associated with the development of novel quality questions on a regular basis, medical education programs may favour the use of item banks. This practice answers the real pragmatic need of having to create exams *de novo* at each administration while benefiting from using psychometrically sound questions to assess students. Unfortunately, programs cannot prevent trainees from engaging in cheating behaviours such as content sharing, and little is known about the impact of re-using items.

**Methods:**

We conducted an exploratory descriptive study to assess the effect of repeated use of banked items within an in-house assessment context. The difficulty and discrimination coefficients for the 16-unit exams of the past 5 years (1,629 questions) were analyzed using repeated measure ANOVAs.

**Results:**

Difficulty coefficients increased significantly (*M* = 79.8% for the first use of an item, to a mean difficulty coefficient of 85.2% for the fourth use) and discrimination coefficients decreased significantly with repeated uses (*M* = 0.17, 0.16, 0.14, 0.14 for the first, second, third and fourth uses respectively).

**Discussion:**

The results from our study suggest that using an item three times or more within a short time span may cause a significant risk to its psychometric properties and consequently to the quality of the examination. Pooling items from different institutions or the recourse to automatic generated items could offer a greater pool of questions to administrators and faculty members while limiting the re-use of questions within a short time span.

## What this paper adds

The re-use of written exam questions answers pragmatic needs in many health professions educational programs. However, this practice puts programs at risk for questions and exam content being leaked from one cohort of examinees to the next cohorts. We examine here the concrete effect on question difficulty and discrimination of re-using written exam questions over a 5-year period. Our results suggest the practice is feasible when limiting the number of times a question is re-used within that time span.

## Introduction

The increased interest in competency-based education (CBE) and programmatic assessment has had an effect on assessment practices within medical education. One of those consequences is the increase in assessment frequency to provide feedback regularly [1–4]. A frequently used and feasible form of assessment to provide feedback opportunities to trainees is the written exams. As such, multiple choice questions (MCQ) and short-answer questions (SAQ) have a prominent role in CBE and programmatic assessment. These and other types of written exam questions may be used to determine progress, identify strengths and weakness in students or to make decisions in terms of success or failure for a unit, course or clerkship rotation [1].

The development of assessment that appropriately and purposefully assesses students’ knowledge and skills, provides feedback, and that yields reliable scores, challenges even the best educators [5–7]. Even more so when one has to limit effects of potential content leakage and thus has to construct new items yearly [8]. The observed increase in assessment frequency is undoubtedly an important additional strain on medical education program administrators and faculty members who may already struggle to develop quality assessment. Thus, the increase in assessment opportunities creates a wicked problem (as defined, for example, in Varpio et al. [9]) for educators and administrators where there is no easy solution when wanting to build-in more assessment opportunities within a program.

To alleviate some of the burden associated with the development of novel quality questions on a regular basis, medical education program administrators and faculty members may favour the use of item banks [10]. Usually, an item bank represents a collection of items or questions used by an organization for their testing or examination purposes. Items in the banks have been previously used and have known psychometric properties such as discrimination and difficulty coefficients [11]. An obvious advantage of item banks is the possibility to use items that have known item properties, only selecting those that meet the highest standards of quality. Consequently, the use of banked items with established psychometric properties could allow undergraduate medical education program administrators and faculty members to control for item quality, overall exam difficulty and hopefully reliability.

The use of banked items creates, however, a risk of item disclosure. The known use of banked items in assessment often leads to strategies such as content sharing [12–14]—between trainees—and are an unfortunate reality in medical education [14]. Content sharing goes beyond the context of written exams to also be observed in the context of performance-based assessment [15] and is per definition detrimental to the validity of assessment data interpretation [16]. Unfortunately, self-reports for cheating behaviours range from 5 to 94% of students adopting some sort of cheating behaviours [14, 17–21]. The creation of unofficial item banks, or braindump sites [12], can lead to a significant proportion of a given test being leaked to future examinees and can consequently impact items’ psychometric properties (questions become easier with exposure and less discriminant) and thus the integrity of future exams [12].

While there are limited studies about the impact of item disclosure in the context of classroom-based assessment, several studies have tackled the issue of item disclosure in the context of repeated exposure to the same items for repeat examinees on certification or licensure exams [10, 22–26]. Most of these studies have shown an increased performance at a subsequent sitting of the test [10, 22–26]. In one study on the topic of repeated questions’ psychometric properties, Wagner et al. [8] concluded that there was little impact of question exposure in the context of a formative assessment, however they lacked the power to observe a statistical difference.

Park and Yang [27] argue against item disclosure in the context of certification and licensure exams because of potential risks to the validity of assessment score interpretation and added onus on exam developers having to develop new questions each year. Given the ubiquitous nature of assessment in health professions education and its increased use in the context of programmatic assessment and CBE, we need empirical data about the impact of re-using written exam questions on their psychometric properties. Classical theory can be used to document psychometric properties; while it may not be as precise and robust as models in the item response theory family it is often used in the context of classroom-based assessment due to the smaller samples [28–30].

The aim of this study was to assess the effect of repeated use of banked end of unit exam questions on their psychometric properties (difficulty and discrimination indices) over a 5-year period in a Canadian undergraduate medical education (UGME) program.

## Methodology

### Study design

We conducted an exploratory and descriptive study to investigate the effect of re-using written exam questions on their psychometric properties. The study was conducted within a Canadian undergraduate medical education program that uses a problem-based learning (PBL) approach. The curriculum has been organized in sequential blocks. During the first two years, students are exposed to 16 separated system-organ blocks in addition to a block dedicated to biomedical knowledge and a block serves as introduction to their future profession as physicians. These blocks are almost exclusively taught through PBL and mobilize 25–35 tutors per block. Each unit has a formal written and oral assessment.

At the time when the study was conducted, the Canadian Tri-Council policy statement on ethical conduct for research involving humans stated that consent was not required from participants for a secondary analysis of anonymized data.

### Data

Written exams for the 16 system-organ PBL blocks within the UGME program are comprised of some banked (i. e., previously used) and some new, MCQ and SAQ. New questions (or significantly modified questions) are written by a faculty member and are subsequently vetted by two faculty members for appropriateness, purposefulness and difficulty (minimal-pass-level cut score). Questions are not pretested. Questions are vetted after each administration, any significant change (e. g., re-phrasing an option) would make that question ‘significantly modified’ and categorized as a new question. There is no washout period for questions, meaning a question could be used two years in a row. Block exams vary in length and ratio of new/used items, as there are no definite standards of practice. More specifically, the written exams can comprise about 50 to 90 questions, with 25 to 60% of re-used questions. MCQ answers receive either 0 or 1 according whether the candidate correctly identifies the appropriate answer. SAQ are marked on a scale of 0 to 1, given a weighted mark, depending on the number of sub-questions answered. For example, if the SAQ requires a list of three diagnoses, each correct diagnosis would be given a 0.33 mark.

We collected data for the 16 different block exams administered to five different cohorts (2007 to 2011 (*n* = 972 students)). In that period, and for all cohorts and exams combined, 1,629 were identified as re-used questions meaning that they were used more than once in that period. These questions were thus included in our analysis. In other words, we pooled data from 90 different exams, with 16 exams administered to each of our five cohorts over the 2.5 years of their pre-clinical training.

Difficulty and discrimination coefficients calculated using the Classical Test Theory were gathered for those 1,629 re-used questions. Although Item Response Theory is recognized for its greater parameter estimate stability, larger sample sizes are required to achieve said parameter invariance [28, 29]. Difficulty and discrimination coefficients, although computed per administration, were calculated on samples of 180 to 200 students which gives the statistical estimation some stability, although it cannot prevent nor correct for group variability as could equating done using IRT modelling. The difficulty coefficients correspond to the mean percentage of candidates who correctly answered the question, and are presented in percentages. A higher value means a higher success rate and thus represents an easy question. The discrimination coefficient represents the corrected point-biserial correlation. Higher values represent more discriminant questions.

### Analysis

Descriptive statistics were computed for difficulty and discrimination coefficients per number of times a question was used. We conducted repeated measure ANOVAs on 1‑ difficulty and 2‑ discrimination coefficients (dependent variable) for the different number of times used (1st, 2nd, 3rd, and 4th) as a within-subject comparison (independent variables). In other words, using the number of times a question was used as a within-subject comparison (where questions are treated as the subjects of interest), we investigated the impact on two dependent variables, that is, the difficulty and the discrimination coefficients. A total of six ANOVAs were computed. Analyses were done using SPSS 21 [31].

## Results

**Fig. 1:**
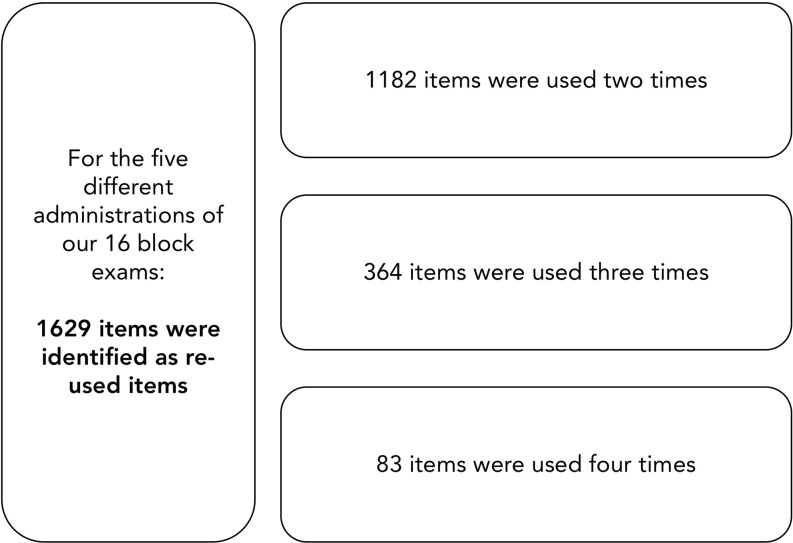
Number of items re-used two-, three-, and four-times in a five year period

Questions were only categorized in one of the three categories (two, three or four uses) as illustrated in Fig. 1. More specifically, our sample included 1,182 questions used twice, 364 questions used three times and 83 questions used four times. Some questions had missing discrimination coefficients (because of difficulty coefficients of 1) which explains the lower sample sizes presented in Tab. 1.

**Table 1:** Average difficulty and discrimination coefficients per number of time questions were used

	Average difficulty (standard deviation)			Average discrimination (standard deviation)		
Number of uses	2	3	4	2	3	4
*N*	1,182	364	83	1,102	349	80
1st use	80.18 (17.69)	79.13 (16.85)	78.06 (18.30)	0.173 (0.114)	0.165 (0.115)	0.184 (0.104)
2nd use	81.69 (17.13)	83.80 (16.18)	80.18 (18.82)	0.171 (0.114)	0.141 (0.111)	0.149 (0.115)
3rd use		84.48 (15.94)	84.31 (14.25)		0.135 (0.107)	0.149 (0.126)
4th use			84.92 (14.77)			0.140 (0.115)
*p* value	<0.0001	<0.0001	<0.0001	0.227	<0.0001	0.004

### Overall difficulty and discrimination coefficients

We observed an increase in question difficulty with repeated use, and independent of the number of uses. The overall mean difficulty coefficient of 79.8% for the first use of a question, to an overall mean difficulty coefficient of 85.2% for the fourth use of a question within a 5-year period (with mean difficulties of 82.2%, and 84.5% for 2nd and 3rd uses respectively). We observed a decrease in discrimination coefficients with repeated use, from an overall mean discrimination coefficient of 0.16 on the first use to an overall mean discrimination coefficient of 0.14 for the last use (*M* = 0.16, and 0.13, for 2nd and 3rd uses respectively).

### Difficulty and discrimination coefficients per number of times the question was used

Tab. 1 more specifically presents the effect of re-using questions on their psychometric properties per number of times a question was used. The increase in question difficulty was significant for questions that were used twice (F[1, 1,181] = 34.444, *p* < 0.001), three times (F[2, 363] = 36.545, *p* < 0.001) or four times (F[3, 82] = 12.989, *p* < 0.001). The decrease in question discrimination was significant for questions that were used three times (F[2, 348] = 15.590, *p* < 0.001) or four times (F[3, 80] = 4.572, *p* < 0.001).

## Discussion and conclusion

The use of in-house item banks answers a real pragmatic need of having to create exams *de novo* at each administration. Re-using items, however, comes with potential disclosure risks which can be a real threat to the validity of assessment data interpretation. This study analyzed the psychometrics properties of 1,629 re-used questions within a 5-year span in the context of a PBL UGME program at a Canadian University.

We observed a change in question property with an increased use of the question. More specifically, items which were re-used over a 5-year period became decreasingly difficult and less discriminating over time. The increase in difficulty coefficients, or the appearance of questions getting easier, may have several underlying contributors. As suggested earlier, item repeat increases the risk of item disclosure and there is evidence in the literature [12–14] for such behaviour. Another potential explanation would be that teachers became better at preparing the students for the examinations. However, the exams in our study were end of PBL blocks tutored by 25–35 different faculty members who do not participate in the exam development which is overseen by one professor per PBL block. Last, differences in item properties, because these coefficients were computed using the Classical Test Theory which is prone to group differences [30], might have been due to real group differences.

Whether the observed change in psychometric properties is pedagogically significant—and can thus impact the validity of assessment data interpretation—is debatable. To assess the pedagogical impact of these results, one should consider what an overall 6% increase in difficulty coefficient and a 0.02 drop in discrimination coefficient represents. In most Canadian universities, the grading system translates a score (number) to a letter with an increase (in qualification) at each 5% increments (e. g., from B to B+ to A−). Thus, creating an exam with frequently re-used banked questions could potentially increase students’ scores and consequently their final grade (which could be an overestimation of their real performance level). The effect of decrease in discrimination may not be as impactful; it seems the discrimination coefficients were only minimally influenced by question re-use.

This study has limitations. The study was conducted within only one program, for a limited time span (5 years), where only a small sample of questions (*n* = 83) was used four times, and no question was used more than four times. We cannot posit on what happens when a question is used more than four times. We pooled data from 90 different exams (18 blocks × 5-year span) and thus had a large sample of re-used questions. Moreover, each exam is comprised of questions written by 2 to 3 different faculty members. We could not access frequency for MCQ options, which also would have been very interesting data to study. If options become less attractive through time, we could speculate about content/questions leakage from one cohort to the next. Although the results of this study cannot generalize beyond similar contexts, the significant changes in psychometric properties should not be overlooked.

Pragmatically, our results suggest that using a question three times or more within a short time span (5 years) can impact the question’s psychometric properties. Pooling questions from different institutions could be an interesting way to increase item-bank size and thus access to questions with known psychometric properties, while limiting the re-use of a specific question within a short time span, as also suggested by Gotzman and colleagues [15]. Gierl and colleagues [32–35] have also proposed, and demonstrated, the potential for automated item generation which could reduce the burden on exam developers to create new questions each year. More specifically, using established parameters (such as patient characteristics and presentation), cognitive models, and a programmed algorithm, users can rapidly generate several exam questions [35, 36]. Some universities, and even licensing authorities, are already engaging in this practice to reduce the burden of creating questions de novo each year. However, pooling questions from different universities and the automatic generation of items should be done with great consideration for differences in learner levels, curriculum and teaching practices.
